# Case report: acute clinical presentation and neonatal management of primary hyperparathyroidism due to a novel CaSR mutation

**DOI:** 10.1186/s12887-018-1319-0

**Published:** 2018-10-30

**Authors:** Manuela Capozza, Iolanda Chinellato, Vito Guarnieri, Natascia Di lorgi, Maria Accadia, Cristina Traggiai, Girolamo Mattioli, Antonio Di Mauro, Nicola Laforgia

**Affiliations:** 1 Neonatology and Neonatal Intensive Care Unit, Department of Biomedical Science ad Human Oncology, University of Bari “Aldo Moro”, Bari, Italy; 2 S.C. Pediatria, P.O.C. SS. Annunziata Hospital, Taranto, Italy; 3 Medical Genetics, IRCCS Casa Sollievo della Sofferenza Hospital, San Giovanni Rotondo, Foggia Italy; 4 Department of Pediatrics, Endocrine, Diabetes and Metabolic Unit, Istituto Giannina Gaslini, University of Genova, Genoa, Italy; 5 Neonatology and Neonatal Intensive Care Unit, Istituto Giannina Gaslini, Genoa, Italy; 6 Pediatric Surgery Unit, Istituto Giannina Gaslini, University of Genoa, Genoa, Italy; 7 Policlinico Hospital, Piazza Giulio Cesare n. 11, 70124 Bari, Italy

**Keywords:** Hypercalcemia, Calcium sensing receptor, Neonatal severe primary hyperparathyroidism, Out-of-hospital cardiorespiratory arrest, Parathyroidectomy

## Abstract

**Background:**

Neonatal severe primary hyperparathyroidism (NSHPT) is a rare autosomal recessive disorder of calcium homeostasis, characterized by striking hyperparathyroidism, marked hypercalcemia and hyperparathyroid bone disease. We report the case of a newborn with a novel homozygous mutation of the CaSR, treated by successful subtotal parathyroidectomy, who had an acute presentation of the disease, i.e. out-of hospital cardiorespiratory arrest. .

**Case presentation:**

A 8-day-old female newborn was admitted to the NICU of University of Bari “Aldo Moro” (Italy) after a cardiorespiratory arrest occurred at home. Severe hypercalcemia was found and different drug therapies were employed (Furosemide, Cinacalcet and bisphosphonate), as well as hyperhydration, until subtotal parathyroidectomy, was performed at day 32. Our patient’s mutation was never described before so that a strict and individualized long-term follow-up was started.

**Conclusions:**

This case of NSHPT suggests that a near-miss event, labelled as a possible case of SIDS, could also be due to severe hypercalcemia and evidentiates the difficulties of the neonatal management of NSHPT. Furthermore, the identification of the specific CaSR mutation provides the substrate for prenatal diagnosis.

## Background

Neonatal severe primary hyperparathyroidism (NSHPT) is a rare autosomal recessive disorder of calcium homeostasis that presents shortly after birth, characterized by striking hyperparathyroidism, marked hypercalcemia and hyperparathyroid bone disease [1].

NSHPT patients shows hypotonia, respiratory distress, bone fractures, intestinal dysmotility and failure to thrive [1] and their neurological development can be significantly affected. If NSHPT is not diagnosed and treated it can be fatal [2]. NSHPT is caused by a loss of function of the calcium-sensing receptor CaSR, whose gene is encoded in the long arm of chromosome 3 (3p-13.3- 21) [3]. More than 250 different CaSR inactivating mutations have been described. The type of mutation can affect both clinical severity and response to treatment. CaSR is a G-protein-coupled receptor found in many tissues throughout the body, but its action is best understood in the parathyroid gland and in the kidney, where parathyroid hormone (PTH) synthesis and secretion and calcium reabsorption and excretion are regulated, respectively [4]. PTH is the main regulator of serum calcium levels and its secretion from the parathyroid is regulated by signals from CaSR [5]. Different treatment options have been proposed for NSHPT, such as drug therapies or surgical removal of the hyperplastic parathyroids [6, 7]. Recently calcimimetics (Cinacalcet) have been used to increase the calcium-responsiveness of mutated CaSR proteins with significant and lasting effect [8]. Medical treatment of NSHPT includes also intravenous hyper-hydration, diuretics and bisphosphonates. However, total or subtotal parathyroidectomy is still the treatment of choice often needed even if very difficult in newborns [9]_._ We report the case of a newborn with NSHPT due to a homozygously inherited mutation not previously described, treated first medically and then by subtotal parathyroidectomy.

### Presenting concerns

A full-term AGA neonate, by third-degree cousins, born at 38 weeks by spontaneous vaginal delivery, after a normal pregnancy, with no significant obstetric and family history was discharged at the 3rd DOL home.

At 8 days of age, because of poor feeding and lethargy, paediatrician was called at home. During her visit, the neonate had a cardiorespiratory arrest. Immediately high quality cardiopulmonary resuscitation (30:2, single rescuer) was initiated and maintained for 3 minutes with reappearance of vital signs. The neonate was immediately brought to the Neonatal Intensive Care Unit of the Department of Biomedical Science and Human Oncology, of “Aldo Moro” University of Bari, Italy .

### Clinical findings

On physical examination, lethargy, hypotonia and absent deep tendon reflexes were evident and a weight loss of 465 g since birth was recorded.. Blood pressure was 68/40 mmHg and SaO_2_ = 97% on room air. Initial laboratory evaluation revealed severe hypercalcemia (serum calcium level = 28.7 mg/dl; RR: 8.6 ± 11.8 mg/dl). High serum alkaline phosphatase activity (663 U/L; RR: 105÷410 U/L), hyperparathyroidism (PTH 465 pg/ml; RR: 6.5÷36.8 pg/ml) and hypophosphatemia (2 mg/dl; RR: 3.1÷7.7 mg/dl) with normal magnesium (2.2 mg/dl; RR 1.8–2.4 mg/dl) and 25-hydroxyvitamin D (33.1 pg/ml; RR: 19.8 ± 79.3 pg/ml) were also found. Urine calcium was 35.8 mg/dl, urine creatinine 15.4 mg/dl with a spot urinary calcium/creatinine ratio of 2.3. During the following days, hypercalcemia persisted: the peak of serum calcium was 29.3 mg/dl with serum-ionized calcium of 14.9 mg/dl (RR: 4.6 ± 5.3 mg/dl).

**Fig. 1:**
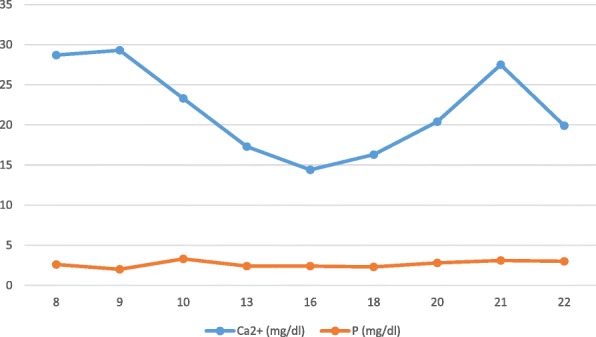
Calcium and phosphate levels from 8 to 22 DOL

The following graph reports calcium and phosphate values in comparison with qualitative data provided (Fig 1).

Total body X-rays revealed generalized skeletal undermineralization and subperiosteal bone resorption. Abdominal, cerebral and cardiac ultrasound were normal. “QT stretching” were found at the ECG as sign of hypercalcemia.

Both parents had calcium levels within normal range.

### Timeline

Table 1 shows the timeline that includes specific dates and times of the patient.

**Table 1:** Timeline

Dates	Relevant Past Medical History and Interventions		
13/11/2016	A full-term AGA neonate, by third-degree cousins, born by spontaneous vaginal delivery, after a normal pregnancy. No remarkable obstetric and family history. Discharged home at the 3rd DOL.		
Dates	Summaries from Initial and Follow-up Visits	Diagnostic Testing (including dates)	Interventions
21/11/2016	During paediatrician’s visit because of poor feeding and lethargy, the neonate had a cardiorespiratory arrest.		High quality cardiopulmonary resuscitation (30:2, single rescuer) for 3 minutes with reappearance of vital signs.
21/11/2016	Admission at NICU-Policlinico of Bari (Italy): significant weight loss, lethargy, hypotonia and absent tendon reflexes.	Severe hypercalcemia (28.7 mg/dl; RR: 8.6 ± 11.8 mg/dl), high serum alkaline phosphatase activity (663 U/L; RR: 105÷410 U/L), hyperparathyroidism (PTH 465 pg/ml; RR: 6.5÷36.8 pg/ml), hypophosphatemia (2 mg/dl; RR: 3.1÷7.7 mg/dl), normal magnesium (2.2 mg/dl; RR 1.8–2.4 mg/dl), normal 25-hydroxyvitamin D (33.1 pg/ml; RR: 19.8 ± 79.3 pg/ml).	Low calcium intake (85 mg/day), intravenous fluid hyperhydration (glucose solution 8% with aminoacids and physiologic solution - 220 ml/kg/day), Furosemide (2 mg/kg/day), Clodronate (1 mg/kg/day): No response (after 7 days of therapy)
26/11/2016	Persistent severe hypercalcemia, polyuria, dehydration, hypotonia, bone changes and failure to thrive.	Total body X-rays (skeletal undermineralization, subperiosteal bone resorption). ECG (anomalies of the recovery phase,“QT stretching”). Abdominal, cerebral and cardiac ultrasound (normal). Parathyroid glands Ultrasound: glands not detectable.	Cinacalcet (0.4 mg/kg/day): No response (after 2 weeks of treatment). No adverse reactions.
05/12/2016	Both parents were clinically and biochemically normal.	Genomic DNA of proband and parents extracted from peripheral blood leukocytes and molecular screening of the Calcium-sensing receptor (CASR) gene (Sanger sequencing).	Proband: a novel autosomal recessive mutation in the CaSR gene: (c.1608 + 1G > A –IVS5 + 1G > A in homozygosity). Parents: presence of the CaSR variant in heterozygosity (FHH clinical state).
13/12/2016	She was transferred to the NICU department of the G. Gaslini Institute of Genova (Italy).	Total calcium level stabilized around 6 mEq/L (equal to 12 mg/dl).	Increasing doses of Cinalcalcet (up to 4 mg/kg/day), low orally calcium intake (45 mg/day), intravenous fluid hyperhydration (up to 220 ml/kg/day).
20/12/2016	She was transferred to the Pediatric Surgery Unit of the G. Gaslini Institute of Genova (Italy).	Parathyroid glands Ultrasound: glands detectable by only the day before surgery.	Subtotal parathyroidectomy: three-excised hyperplastic parathyroid glands (5 × 3, 1.5 × 1.4 and 1.4 × 1.2 mm, respectively).
22/12/2016	Two days after surgery: transient asymptomatic hypocalcemia appeared (hungry bone syndrome).	X-rays of the right arm: pathological fracture of the humerus neck.	Daily Calcium gluconate replacement (0,5 ml/kg) and α-Calcidol (0,05 mcg/kg) for 40 days.
31/01/2017	Slow and progressive improvement of clinical conditions and hypocalcaemia.		Stop-therapy 40 days post-surgery (Calcium gluconate and α-Calcidol).
	A longer and personalized follow up started in order to confirm if the partial parathyroidectomy was sufficient for calcium balance or a second surgical approach is needed.		

### Diagnostic focus and assessment

The differential diagnosis for hyperparathyroidism is wide and includes parathyroid adenoma/hyperplasia, PTH-like peptide secondary to malignancy, CaSR mutation, PTH receptor mutation and Vitamin D excess.

Considering the early onset, life-threatening hypercalcemia and the biochemical findings in the patient, genetic studies of CASR gene were performed revealing NSHPT secondary to a CaSR gene mutation. NSHPT is often discovered during the first days of life because of severe, symptomatic, PTH-dependent hypercalcemia, polyuria, dehydration, hypotonia, bone changes and failure to thrive. Untreated NSHPT can be letal or have devastating neurodevelopmental effects [10]_._

CaSR is a seven transmembrane G-protein coupled receptor essential for normal calcium homeostasis, located in organs involved with calcium metabolism, such as the parathyroid cells, the parafollicular thyroid-C cells, the kidney epithelium and cells in bone and intestine [11]_._ It is encoded by chromosome 3p-13.3- 21 loci [12]. Heterozygous mutations of CaSR gene cause mild forms of asymptomatic hypercalcemia while homozygous mutations cause a rare form of NSHPT [13]_._

After written informed consent of the parents, genomic DNA of proband and parents was extracted from peripheral blood leukocytes and molecular screening of the 7 exons including intron-exon boundaries of the CASR gene was performed as previously described [14] at the Medical Genetics, IRCCS “Casa Sollievo della Sofferenza” Hospital in San Giovanni Rotondo, Foggia, Italy.

**Fig. 2:**
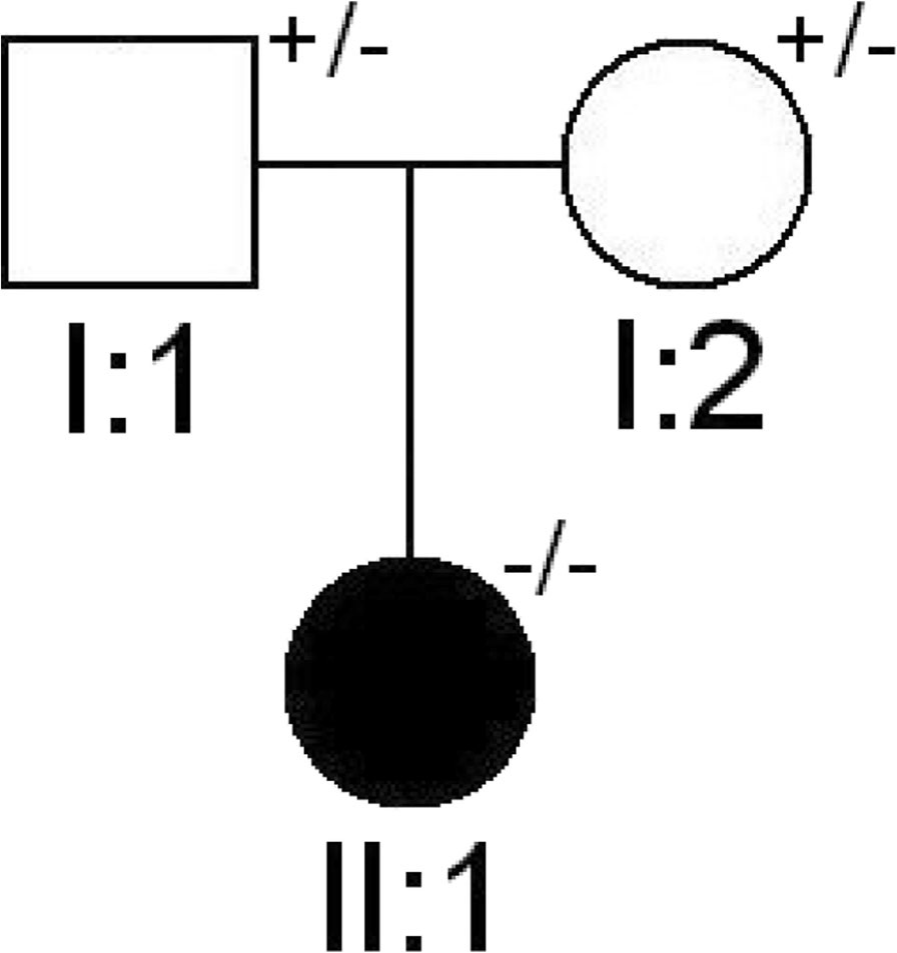
Electropherograms showing the heterozygous mutation in the father and mother and the homozygosity in the proband. On the bottom the control

NSHPT was confirmed by the finding of a novel autosomal recessive mutation of the CaSR gene, causing the loss of receptor’s function. Sequence analysis revealed a previously undefined mutation of the donor splicing site of the intron 5, namely c.1608 + 1G > A –IVS5 + 1G > A, in homozygosity (Fig. 2).

**Fig. 3:**
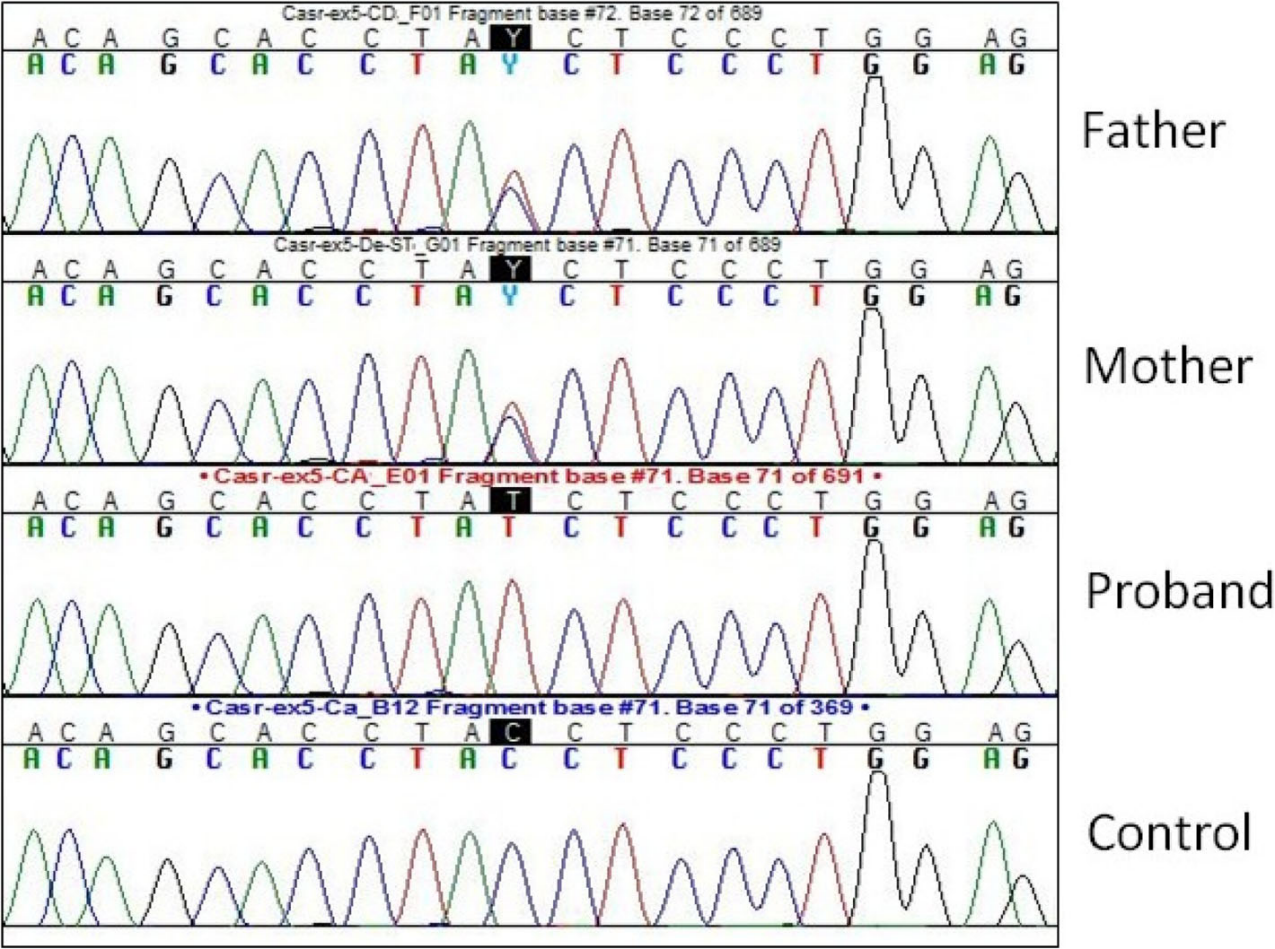
Pedigree of the family under study. Filled symbol indicates the affected proband. Clear symbols indicate the unaffected status; +/− = presence/absence of the mutation

Segregation analysis of parents evidentiated CaSR variant in heterozygosity, confirming the autosomal recessive inheritance of NSHPT and the familial hypocalciuric hypercalcemia (FHH) clinical state of the parents, although asymptomatic (Fig. 3).

### Minigene-based functional approach

The CASR gene is expressed in the parathyroids, bone, kidneys and neural cells but it is not expressed into the blood. Thus, in absence of a source of these tissues, we verified the mutation by an in-vitro assay that artificially reproduced the splicing process on a chimeric gene. The presumed altered mRNA splicing of this novel variant was tested by an “in vitro minigene-based assay” [14]. Briefly, the exon 5 of the CaSR gene along with 150 intronic flanking bases was PCR amplified with primers containing the HindIII and XhoI restriction sites. The amplicon was cloned into the HindIII/XhoI digested pCDNA3.1 vector containing a “chimeric” genetic sequence between the exon 1 of the PTHR1 gene and the exons 2 and 3 of the RAB1 gene. Sequencing of the vector confirmed the correctness of the construct that was further modified in order to introduce the splicing mutation (IVS5 + 1G > A) by a site-specific mutagenesis approach [14]. Both the vectors (hereafter named Mini-WT and Mini-Mut) were transfected in (700 thousands) HEK293 cells seeded in six well plates with Fugene Transfection reagent (Promega, Madison, WI, USA), following manifacturer’s instructions. After 48 h, RNA was extracted with Trizol (Qiagen, Hilden, Germany), retrotranscribed with the Superscript III (ThermoFisher, Whaltam, MA, USA), PCR amplified with specific primers annealing on the chimera gene (mutagenesis and PCR primer sequence, annealing temperatures and size of the amplicons are available upon request). Finally amplicons were loaded on an ethydium bromide (1%, Sigma Aldrich) stained 1,5% agarose gel; they were purified (ExoSap-IT, ThermoFisher, Whaltam, MA, USA) and directly sequenced (Big Dye Terminator Cycle Sequencing Kit v.1.1, ThermoFisher, Whaltam, MA, USA).

**Fig. 4:**
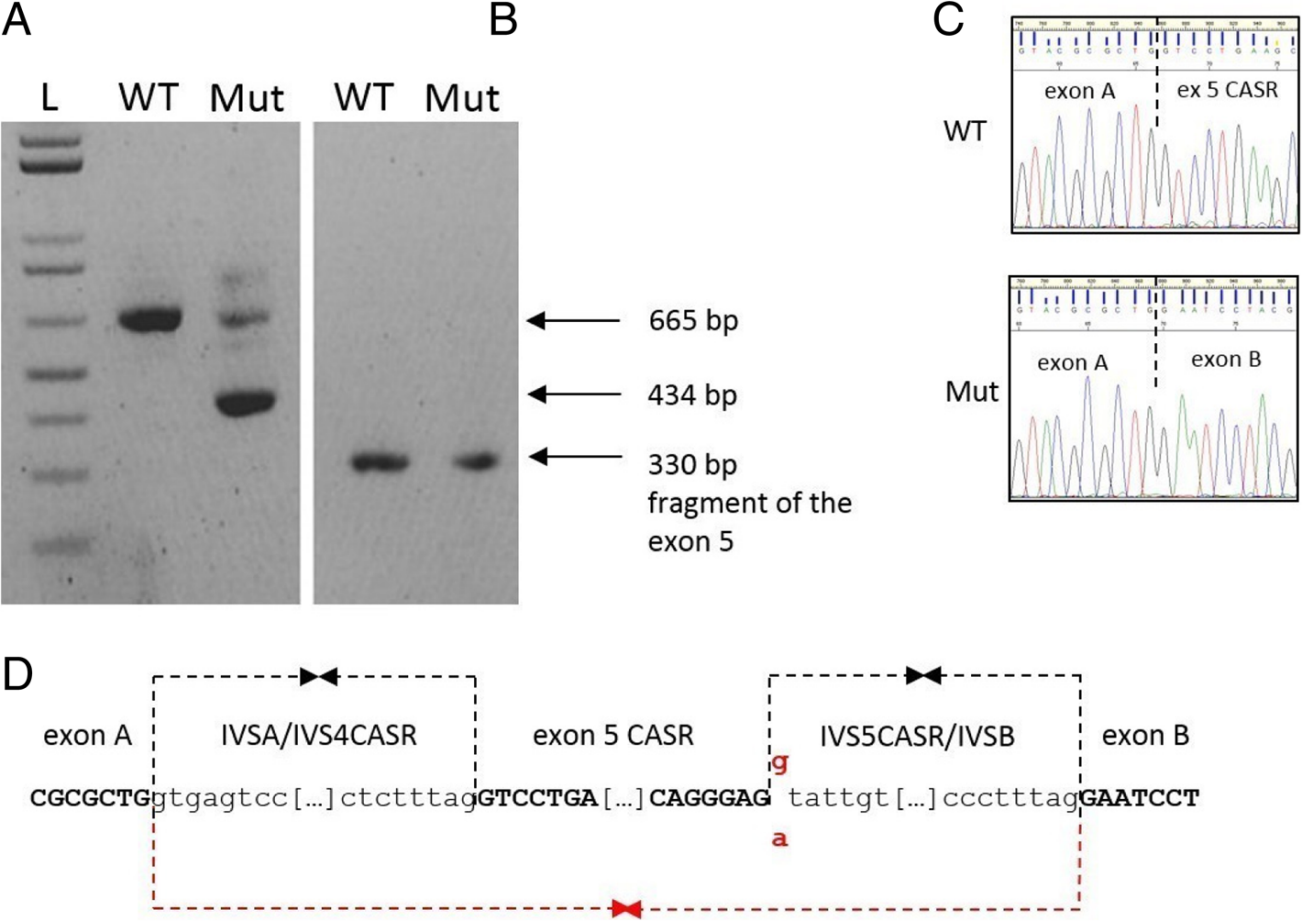
Schematic showing the effect of the splicing mutation. **a**. The retrotranscription of RNA extracted from HEK293 cells transfected with WT/Mutated minigene vectors (see the methods) leads to amplicons of different size: a full chimeric amplicon (665 bp) and a shorter chimeric cDNA fragment (434), both sequenced in **c**; **b**. cDNA amplification with an internal primer designed on the exon 5, shows that both the WT and the mutated RNA produce a normal amplicon (here not quantified): this is partially confirmed by the “faint” band in the lane of mutated cDNA; **d**: diagram showing the exon skipping mechanism induced by the presence of the IVS5 + 1G > A mutation

The mini-gene assay proved the complete skipping of the exon 5 of the CaSR gene (Fig. 4a-c-d), thus leading to an in-frame deletion of 77 residues and thus to a shorter, but presumably expressed, receptor.

Moreover, a different reverse primer specifically designed on the 3′ end of the exon 5, was used to amplify the WT and mutated cDNAs. In both the cases, an amplicon was obtained thus proving that the mutated vector was able to read-through the splicing mutation, giving a (not quantified) amount of WT CaSR RNA form (Fig. 4b). This is can be also confirmed by the higher “faint” band present in the lane of mutated cDNA, having the same size of the correct WT cDNA amplicon (Fig. 4).

### Therapeutic focus and assessment

For the management of hypercalcemia, the newborn received very low calcium intake, intravenous hyper-hydration (total intake 220 ml/kg/day) and Furosemide (2 mg/kg/day), without significant reduction of calcium levels. Ten days after, Cinacalcet (0,4 mg/kg/day), a type 2 calcimimetic agent that increases CaSR affinity for calcium, leading to parathormone suppression and increasing calcium renal excretion, after a written informed consent obtained from his parents, was started [15]. She initially responded, but 10 days after start therapy serum calcium raised up to 27.5 mg/dl, with a serum ionized calcium of 14.9 mg/dl. Bisphosphonates (Clodronate 1 mg/kg for 3 days) were then associated to Cinacalcet and Furosemide [16], but hypercalcemia did not resolve.

Finally, at 1 month of life, she was transferred to the NICU department of the Giannina Gaslini Institute, Genova, Italy, where the child was stabilized to a total calcium level around 12 mg/dl with increasing doses of Cinalcalcet (up to 4 mg/kg/day), low orally calcium/free vitamin D formula, and hyper-hydratation before undergoing an exploratory surgery of the neck. Parathyroid glands were repeatedly undetectable by ultrasound up to the day before surgery. Subtotal parathyroidectomy was then performed with removal of three (two left and one right) hyperplastic parathyroid glands (5 × 3, 1.5 × 1.4 and 1.4 × 1.2 mm, respectively). Baseline PTH level was 1339 pg/ml and intraoperative PTH by bilateral jugular sampling of blood demonstrated 30 min after excision a bilateral PTH reduction of 75% from baseline, and at 70 min a further reduction on the left side (202 pg/ml) and an increase on the right side (658 pg/ml). The surgeons looked for any other areas of the neck region excluding any visible nodes.

It is not yet clear whether subtotal parathyroidectomy or total parathyroidectomy with reimplantation would be best. Some studies highlight they have similar rates of complications, readmission, and 30-day mortality, but subtotal parathyroidectomy is less likely to have extended hospital stay. [17]

### Follow-up and outcomes

Two days after surgery, the patient developed transient asymptomatic hypocalcemia (Ca^2+^ 5 mg/dl) that required daily Calcium gluconate replacement (0,5 ml/kg) and α-Calcidol (0,05 mcg/kg) for 40 days. The X-rays of the right arm showed fracture of the humerus neck and severe osteoporosis.

Hypocalcemia is a common problem after parathyroidectomy because of reduction in bone reabsorption and increase in bone formation. The hypocalcemia is generally transient but it could be severe and prolonged because of a chronic increase in bone resorption induced by previous high levels of PTH (“hungry bone syndrome”) [18].

One month post-surgery, her calcium and α-Calcidol requirements decreased, and 10 days later, these medications were suspended.

She began the follow up with controls of serum calcium levels, which at the time were always normal. We believe that in our NSHPT case, due to a novel homozygous inherited intronic mutation of the CaSR, a longer and personalized follow up is warranted in order to confirm if the partial parathyroidectomy was sufficient for calcium balance or a second surgical approach is needed to remove the fourth gland or any residual.

## Discussion

NSHPT is a rare life-threatening disease characterized by marked hypercalcemia, diffuse parathyroid hyperplasia and skeletal demineralization, due to the biallelic inactivation (homozygous or compound heterozygous mutations) of the CaSR gene.

NSHPT-related mutations have been identified throughout the coding sequence and result in PTH-hyperproduction. The overproduction of PTH leads to hypercalcemia and hypocalciuria due both to significant calcium release from bones and increase of renal calcium reabsorption [19]. De novo heterozygous missense mutations, compound heterozygous missense mutations, frameshift and nonsense mutations have been reported in patients with NSHPT [3, 20, 21].

Our patient shows a homozygous novel mutation of the splicing donor site of the intron 5 in homozygosity. Both asymptomatic FHH parents were found to be heterozygous for this novel mutation, suggesting an autosomal recessive pattern of transmission.

In vitro functional assay proved that this novel mutation leads to an altered splicing process with the skipping of the exon 5, thus presumably giving a short CASR protein, lacking 77 residues of the extracellular domain, previously reported to be a not functional but normally expressed in keratinocytes [22]. Interestingly, it has been proved that this alternatively spliced transcript has a dominant-negative effect on the wild type copy making it less sensible to calcium concentrations and inducing its decrease during keratinocyte differentiation [22]. However, it has to be noted, that this alternative transcript naturally occurs in specified peripheral tissues, in absence of any mutational event. In our clinical case, a different landscape of the mutation could be deduced. We provided evidence that the splicing machinery is able to read through the mutation to give an amount of correct transcript that, however, at this stage, we were not able to quantify. We can hypothesize that in the proband, homozygous for this mutation, this amount of correct mRNA was not enough to compensate the overall huge loss of protein function. In the parents, who already had the 50% of WT protein, the presence of this additional amount of correct mRNA coming from the mutated allele, may have contributed to making them asymptomatic. However, although our results have been obtained “in vitro” and with a cellular system that could not reproduce entirely the in vivo state, our hypothesis would explain why the parents are clinically and biochemically normal. Further studies on mRNAs extracted from the fibroblasts of the parents and proband would clarify this issue.

Moreover, we confirm that the use of Cinacalcet could be indicated provided that the CASR mutation does not induce a premature truncation or a structural conformation change of the protein. In our case, although in absence of a specific functional proof, but taking into account of the positive effect of the Cinacalcet in the first weeks of treatment, we might presume that the deletion of 77 residues located in the ECD did not compromise the binding of the drug with the ICD. In our patient, a trial of Cinacalcet was initiated at 20 days of life resulting in escaping responses of calcemia with increasing doses of calcimimetic [23]. This is consistent with Soblechero et al. that reported a case of neonatal hypercalcemia due to a homozygous deletion/frameshift mutation in the *CaSR* in which there was therapeutic failure of Cinacalcet [1]. The success or failure in response to therapy with Cinacalcet can depend on the CASR genotype [24]. In all cases of NSHPT, a trial with Cinacalcet could be attempted but, if ineffective, definitive surgical treatment is necessary. If not treated by parathyroidectomy, fully developed NSHPT can have a devastating effect on the psychomotor development and it can be fatal, because of complications of hypercalcemia [3]. Respiratory failure, extreme hypercalcemia or failure to thrive are all indications for early total or subtotal parathyroidectomy, which can significantly improve prognosis and quality of life of infants with NSHPT.

## Conclusions

The management of patients with NSHPT can be very challenging. This case of NSHPT suggests that a near-miss event, as well as a case of SIDS, could be due to severe hypercalcemia and also presents the difficulties of medical management.

The identification of the specific CaSR mutation is useful not only for prenatal diagnosis, but also because could explain the response to Cinacalcet.

In most cases, surgical treatment remains the gold standard treatment of NSHPT. There are cases of subtotal parathyroidectomy followed for 10 years of follow-up, without need for a second surgical treatment [25].

## Abbreviations

AGA: Appropriate for gestational age
CaSR: Calcium sensing receptor
DOL: Day of life
FHH: Familial hypocalciuric hypercalcemia
NSHPT: Neonatal severe primary hyperparathyroidism
PTH: Parathyroid hormone.
