# Correction to: Case report: acute clinical presentation and neonatal management of primary hyperparathyroidism due to a novel CaSR mutation

**DOI:** 10.1186/s12887-019-1850-7

**Published:** 2019-11-27

**Authors:** Manuela Capozza, Iolanda Chinellato, Vito Guarnieri, Natascia Di Iorgi, Maria Accadia, Cristina Traggiai, Girolamo Mattioli, Antonio Di Mauro, Nicola Laforgia

**Affiliations:** 1 Neonatology and Neonatal Intensive Care Unit, Department of Biomedical Science ad Human Oncology, University of Bari “Aldo Moro”, Bari, Italy; 2 S.C.Pediatria, P.O.C. SS. Annunziata Hospital, Taranto, Italy; 3 Medical Genetics, IRCCS Casa Sollievo della Sofferenza Hospital, San Giovanni Rotondo, Foggia, Italy; 4 Department of Pediatrics, Endocrine, Diabetes and Metabolic Unit, Istituto Giannina Gaslini, University of Genova, Genoa, Italy; 5 Neonatology and Neonatal Intensive Care Unit, Istituto Giannina Gaslini, Genoa, Italy; 6 Pediatric Surgery Unit, Istituto Giannina Gaslini, University of Genoa, Genoa, Italy; 7 Policlinico Hospital, Piazza Giulio Cesare n. 11, 70124 Bari, Italy

**Correction to: BMC Pediatrics (2018) 18:340**

**https://doi.org/10.1186/s12887-018-1319-0**

In the published article [1], an error has been noticed in the author group section.

Namely, one of the author names is captured incorrectly: Natascia Di lorgi (using lowercase letter L in family name) should be spelled as Natascia Di Iorgi (using uppercase letter I).
